# Translational profiling reveals novel gene expression changes in the direct and indirect pathways in a mouse model of levodopa induced dyskinesia

**DOI:** 10.3389/fncel.2024.1477511

**Published:** 2025-03-12

**Authors:** Sabika Jafri, Mahdi Ghani, Natalie Stickle, Carl Virtanen, Lili-Naz Hazrati, Naomi P. Visanji

**Affiliations:** ^1^Krembil Brain Institute, University Health Network, Toronto, ON, Canada; ^2^Tanz Centre for Research in Neurodegenerative Disease, University of Toronto, Krembil Discovery Tower, Toronto, ON, Canada; ^3^University Health Network Microarray Centre, Toronto Medical Discovery Tower, Toronto, ON, Canada; ^4^Department of Pathology, McGill University, Montreal, QC, Canada; ^5^Department of Laboratory Medicine and Pathobiology, University of Toronto, Toronto, ON, Canada

**Keywords:** Parkinson's disease, levodopa, dyskinesia, gene expression, MAPK pathway

## Abstract

**Introduction:**

The molecular mechanisms underlying L-dihydroxyphenylalanine (LDOPA) induced dyskinesia in Parkinson's disease are poorly understood. Here we employ two transgenic mouse lines, combining translating ribosomal affinity purification (TRAP) with bacterial artificial chromosome expression (Bac), to selectively isolate RNA from either DRD1A expressing striatonigral, or DRD2 expressing striatopallidal medium spiny neurons (MSNs) of the direct and indirect pathways respectively, to study changes in translational gene expression following repeated LDOPA treatment.

**Methods:**

6-OHDA lesioned DRD1A and DRD2 BacTRAP mice were treated with either saline or LDOPA bi-daily for 21 days over which time they developed abnormal involuntary movements reminiscent of dyskinesia. On day 22, all animals received LDOPA 40min prior to sacrifice. The striatum of the lesioned hemisphere was dissected and subject to TRAP. Extracted ribosomal RNA was amplified, purified, and gene expression was quantified using microarray.

**Results:**

One hundred ninety-five significantly varying transcripts were identified among the four treatment groups. Pathway analysis revealed an overrepresentation of calcium signaling and long-term potentiation in the DRD1A expressing MSNs of the direct pathway, with significant involvement of long-term depression in the DRD2 expressing MSNs of the indirect pathway following chronic treatment with LDOPA. Several MAPK associated genes (*NR4A1, GADD45G, STMN1, FOS*, and *DUSP1*) differentiated the direct and indirect pathways following both acute and chronic LDOPA treatment. However, the MAPK pathway activator *PAK1* was downregulated in the indirect pathway and upregulated in the direct pathway, strongly suggesting a role for *PAK1* in regulating the opposing effects of LDOPA on these two pathways in dyskinesia.

**Discussion:**

Future studies will assess the potential of targeting these genes and pathways to prevent the development of LDOPA-induced dyskinesia.

## 1 Introduction

Since its first use in the early 1960's, dopamine replacement, using the dopamine precursor L-dihydroxyphenylalanine (LDOPA), has remained the most effective therapy for the motor symptoms of Parkinson's disease (PD). However, following long-term treatment with LDOPA, >90% of PD patients develop highly debilitating abnormal involuntary movements termed LDOPA induced dyskinesia (LID) (Obeso et al., 2000; Fabbrini et al., 2007; Rascol, 2000; Fabbrini and Guerra, 2021). In 2014 a Priority Setting Partnership, commissioned by Parkinson's UK, identified LID as 3rd of 96 unmet needs in PD (Deane et al., 2015). The socioeconomic impact of LID places an enormous burden on the patient, caregiver and healthcare system (Dodel et al., 2001), however, as the pathogenesis of LID is poorly understood, treatment options are very limited (AlShimemeri et al., 2020). Once developed, LID is virtually impossible to reduce or reverse and sensitivity to developing dyskinesia on re-exposure persists even after long periods of treatment discontinuation.

Behavioral sensitization to repeated LDOPA treatment is also seen in animal models of PD (Bezard et al., 2001; Lundblad et al., 2002, 2005, 2004; Angela Cenci and Lundblad, 2007; Jenner, 2008). In the 6-hydroxydopamine (6-OHDA) lesioned rodent, sensitization to repeated LDOPA treatment leads to abnormal involuntary movements (AIMs) reminiscent of LID. These rodent behaviors have been shown to have a similar pharmacology to LID in humans (Lundblad et al., 2005). Extensive studies in these animal models have revealed that dopamine can directly modulate the functioning of the striatum and that the pathophysiology of LID involves a wide range of changes in the basal ganglia which have been characterized using a variety of methods. These studies have found that both presynaptic and postsynaptic mechanisms are important in governing the manifestation of LID, with changes in the regulation of several genes as well as alterations in the electrophysiological activities of striatal circuits and synaptic plasticity (Huot et al., 2013; Spigolon and Fisone, 2018; Mosharov et al., 2015; Surmeier et al., 2014; Borgkvist et al., 2018; Hutny et al., 2021).

Overstimulation of postsynaptic dopamine receptors located on GABAergic medium spiny neurons (MSNs) in the dorsal striatum of the basal ganglia have been shown to be the site of the initial generation of LID (Fisone and Bezard, 2011). The MSNs form two distinct projection pathways, exerting opposing effects on motor activity: the direct DRD1A dopamine receptor expressing pathway projects to the substantia nigra pars reticulata and medial globus pallidus and promotes locomotion, whereas the indirect DRD2 dopamine receptor expressing pathway projects to the lateral globus pallidus and inhibits locomotion (Albin et al., 1989; Alexander and Crutcher, 1990). To fully understand how dopaminergic stimulation at the level of the MSNs leads to the development of LID, it is vital to study the molecular responses of these two distinct populations of MSNs to LDOPA in isolation (Cenci et al., 2018; Ryan et al., 2018). However, striatal MSNs are both anatomically intermixed and morphologically indistinguishable (Calabresi et al., 2014), thus an inability to adequately discriminate the MSNs of the direct and indirect pathways has been a significant roadblock to the full understanding of the development of LID. In the present study, and as we have previously, we have made use of two transgenic mouse lines combining translating ribosomal affinity purification (TRAP) with bacterial artificial chromosome (Bac) technology to purify RNA from either DRD1A expressing striatonigral MSNs or DRD2 expressing striatopallidal MSNs (Heiman et al., 2008, 2014b; Visanji et al., 2015; Dougherty, 2017). Bac mice that express enhanced green fluorescent protein (EGFP) under the control of either the DRD1A or DRD2 receptor promoter were originally developed as part of a groundbreaking initiative led by the National Institute of Neurological Disorders and Stroke Gene Expression Nervous System Atlas project (Heintz, 2001; Gong et al., 2003; Gerfen et al., 2013). These mice express EGFP in the same spatiotemporal pattern as the endogenous DRD1A or DRD2 protein. Thus, visualization of EGFP expression confirmed that DRD1A-bac mice selectively express EGFP in the medial globus pallidus and Substantia nigra pars reticulata (the terminal fields of the direct pathway) and DRD2-bac mice selectively express EGFP in the lateral globus pallidus (the terminal field of the indirect pathway) (Valjent et al., 2009; Gerfen, 2006; Thibault et al., 2013). Since their advent these innovative Bac mice have been employed in a wide range of studies requiring the segregation of striatal MSNs of the direct and indirect pathways (Valjent et al., 2009; Thibault et al., 2013). Subsequently, Heiman et al. combined these bac lines with their innovative TRAP methodology to generate BacTRAP mice that express an EGFP tagged L10a ribosomal subunit under the control of either the DRD1A or DRD2 receptor promoter, resulting in the expression of EGFP-L10a in the MSNs of either the direct or indirect pathway, respectively (Heiman et al., 2008; Emery and Barres, 2008). Characterization of the expression of EGFP-L10a in these DRD1A and DRD2 BacTRAP lines confirmed the selective expression of EGFP in the MSNs of the direct and indirect pathway terminal fields respectively (Heiman et al., 2008). In addition, striatal EGFP in DRD2 BacTRAP mice was shown to colocalize with enkephalin, a well established marker for MSNs of the indirect pathway whereas in DRD1A BacTRAP animals there was no colocalization with enkephalin (Heiman et al., 2008). The EGFP-L10a subunits of the MSNs of both the direct and indirect pathways can be purified along with the associated mRNA which can then be profiled using microarray to reveal differences in translational RNA expression in these two distinct cell populations. Indeed, translational profiling in these two mouse lines has previously identified >70 transcripts enriched in the indirect pathway and >150 transcripts enriched in the direct pathway, as well as validating the differential expression of known markers that distinguish these two cell populations (Heiman et al., 2008).

Here, we employ a widely used model of LID in 6-OHDA lesioned rodents, using two transgenic mouse lines to selectively identify changes in translational gene expression induced by chronic LDOPA in either DRD1A expressing striatonigral (direct pathway) or DRD2 expressing striatopallidal (indirect pathway) MSNs and reveal mechanisms underlying LID that might aid the development of preventative strategies for one of the major challenges in the treatment of PD.

## 2 Methods

### 2.1 Transgenic mice

All animal use was in accordance with approved local institution protocol and the regulations defined by the Canadian Council on Animal Care. Two Bac transgenic mouse lines were obtained from The Rockefeller University, New York, NY, USA. Both transgenic mouse lines express an EGFP-L10a fusion protein either under the control of the DRD1A (line CP73) or DRD2 (line CP101) promoter. For a full description of the mice, please refer to Doyle et al. (2008). Both lines were on a C57BL/6J/Swiss-Webster background and were maintained as transheterozygous. Our study comprised 4 experimental groups based on two factors; MSN type (Drd1 EGFP-L10a expressing, DRD2 EGFP-L10a expressing) and LDOPA treatment (acute or chronic). The number of animals in each group was as follows: Acute LDOPA Drd1a (7), Acute LDOPA Drd2 (8), chronic LDOPA Drd1a (4), chronic LDOPA Drd2 (4). The number of sample replicates was 1.

### 2.2 6-hydroxydopamine (6-OHDA) lesioning of the median forebrain bundle (MFB)

All animals were lesioned at 35 days of age. Thirty minutes prior to lesioning, animals received desipramine (25 mg/kg) and pargyline (5 mg/kg) intraperitoneally (i.p.) (Both Sigma Aldrich). Under anesthesia (isoflurane), 6-OHDA (3 μg in 0.6 μl) (Sigma Aldrich) was infused unilaterally into the medial forebrain bundle (MFB) at a flow rate of 0.2 μl/min at the following coordinates from Bregma: AP −1.2, ML −1.1, DV −5.0 mm, according to the mouse brain atlas (Paxinos and Franklin, 2013). The needle was left *in situ* for 5 min before being retracted. Animals had a 14-day recovery period during which time they were administered 3 ml/day lactated ringers containing 5% dextrose and kept on heat pads until they were able to maintain a stable body weight. Fourteen days post-lesion, all animals were assessed for rotational bias by measuring spontaneous full rotations contraversive and ipsiversive to the lesioned hemisphere over a 10 min period in glass cylinders. Post-mortem, lesion efficiency was assessed by calculating nigral tyrosine hydroxylase (TH) immunoreactive cell loss. Following fixation in 4% paraformaldehyde, blocks encompassing the entire midbrain were embedded in paraffin and 5 μm-thick serial sections were taken from −3.08 and −3.28 mm relative to bregma, according to the mouse brain atlas (Paxinos and Franklin, 2013). Briefly, endogenous peroxidase was blocked with 3% hydrogen peroxide, antigen retrieval was done using 10 mM citrate buffer at pH 6.0, and sections were stained overnight with rabbit polyclonal antibody to TH (Novus Biologicals) at 1/1,500 dilution. The staining was finished with Vector's Peroxidase ImmPRESS detection system, the color was developed by 3,3′-Diaminobenzidine, and sections were counterstained with Mayer's hematoxylin prior to being coverslipped. TH positive cells were counted manually and defined as those with a brown cell membrane and distinct lighter rounded cell body.

### 2.3 LDOPA treatment and abnormal involuntary movement (AIMS) analysis

All animals were administered either vehicle, or LDOPA methyl ester/benserazide (6/15 mg/kg; Sigma Aldrich) i.p. twice daily (>6 h apart) for 21 days. On days 1, 7, 14 and 21 of treatment, animals were assessed for abnormal involuntary movements (AIMs). Immediately post treatment, animals were placed in single cages and their behavior was observed for 1 min every 20 min for a period of 2 h. On day 22, animals were observed for a period of 1 min 40 min post treatment with LDOPA/benserazide (6/15 mg/kg). The AIMs scale, as described in detail by Angela Cenci and Lundblad (2007), was used to assess the level of dyskinesia. Briefly, there were three categories of scored AIMs: axial, limb, and orofacial. Each category was rated on a scale of 1–4 based on the maximum severity of behavior observed in each 1 min period. Please refer to Supplementary Table 1 for a full description of each AIMs category and rating.

### 2.4 Translating ribosome affinity purification (TRAP)

On day 22, all animals were sacrificed 40 min post treatment with LDOPA/benserazide (6/15 mg/kg). Mice were killed by cervical dislocation and the striatum of the lesioned hemisphere was quickly dissected on ice. TRAP was performed and analyzed from each individual mouse striatum. Dissected striata from each mouse were individually homogenized, using a motor driven Teflon-glass homogeniser, in ice cold lysis buffer (20 mM HEPES KOH, 5 mM MgCl_2_, 150 mM KCL, 0.5 mM DTT, 100 μg cyclohexaminde, 40 U/ml Rnasin and protease inhibitor cocktail, pH 7.4). A post-nuclear supernatant was prepared by centrifugation at 2,000*g* at 4°C for 10 min to which 1% NP-40 (EMD Millipore, Billerica, Massachusetts) and 30 mM 1,2-diheptanoyl-sn-glycero-3-phosphocholine (DHPD, Avanti Polar Lipids, Alabaster, AL) was added. Finally, a post mitochondrial supernatant was prepared by centrifugation at 20,000*g* at 4°C for 10 min. Striatal homogenates were immunoprecipitated by end-over mixing for 30 min at 4°C with Dynal protein G magnetic beads (Invitrogen, Carlsbad, CA) pre-coated with 50 μg each of two anti-GFP antibodies (clones 19C8 and 19F7, Memorial Sloane Kettering, NYC, NY). Following immunoprecipitation, beads were collected using a magnetic rack and washed four times with an ice-cold high salt buffer (20 mM HEPES-KOH, 5 mM MgCl_2_, 350 mM KCL, 0.5 mM DTT, 1% NP-40, 100 μg cyclohexaminde, pH 7.4). RNA was extracted from the beads and purified according to the manufacturer‘s instructions using an Absolutely RNA nanoprep kit (Stratagene, La Jolla, CA) with in column DNA digestion and frozen prior to gene expression profiling being performed. The quantity of RNA was determined using a Nanodrop 1000 spectrophotometer (Wilmington, DE) and the quality was determined using an Agilent 2100 Bioanalyzer (Foster City, CA).

### 2.5 Microarray

One nanogram of extracted RNA from each striatal sample (Stratagene/Agilent) was amplified using the WT-Ovation Pico RNA amplification System Version 1.0 (Nugen) and cDNA was run on a bioanalyzer (Agilent) for quality control. All samples had a RIN number of >6.3. 3.5 μg cDNA was biotin labeled (Nugen Illumina) 1.5 μg of cDNA was hybridized to the Illumina Mouse WG-6 V2.0 BeadChip containing 45,821 probes. BeadChips were incubated at 48°C with a rotation speed of 5 for 18.0 h of hybridization. BeadChips were then washed and stained as per Illumina protocol and scanned on the iScan (Illumina). Data files were quantified in GenomeStudio Version 2010.2 (Illumina). All samples passed Illumina's sample dependent and independent quality control metrics. Data were further checked for overall quality using R (v2.14.1) with the Bioconductor framework and the LUMI package installed. There were no discernible outliers.

### 2.6 Pathway analysis

Enrichr (http://amp.pharm.mssm.edu/Enrichr/) was applied to identify significant Kyoto Encyclopedia of Genes and Genomes (KEGG) defined pathways associated with the set of differentially expressed genes in each experimental group.

### 2.7 Quantitative real-time RT-PCR

Remaining cDNA from the amplified product used for microarray was used for RT-PCR. PCR was performed using Promega GoTaq qPCR mastermix according to the manufacturer's instructions (Promega, Madison, WI). Each reaction comprised 1 ng cDNA, 12.5 μl GoTaq and 0.2 μM final concentration of each primer. Cycling and detection were carried out using an Applied Biosystems 7500 Real Time PCR System and data quantified using Sequence Detection Software Version 1.4 (Applied Biosystems, Carlsbad, CA). PCR was performed for a total of 40 cycles (95° for 15 s, 60° for 60 s) followed by a dissociation stage. Each sample was assayed in duplicate. All data were normalized to Actin and quantification was carried out via the absolute method using standard curves generated from pooled cDNA representative of each sample to be analyzed. Please refer to Supplementary Table 2 for a complete list of all primers used in the present study.

### 2.8 Statistical analysis

#### 2.8.1 Gene expression analysis

Data was imported into Genespring v12v12.0 (Agilent) for analysis and normalized with a quantile normalization followed by a median centering. All data analysis and visualization were performed on log_2_ transformed data. There were 4 groups overall split into two factors: MSN type (Drd1 EGFP-L10a expressing, DRD2 EGFP-L10a expressing) and LDOPA treatment (acute or chronic). Data was first filtered to remove the confounding effect probes that show no signal may have on subsequent analysis. Only probes that were in the upper 80th percentile of the distribution of intensities in 100% of any of the one of four above groups were allowed to pass through this filtering. The final set for analysis contained 32,362 probes. To find those genes that statistically varied with the greatest confidence between sample groups, a one-way ANOVA with a Benjamini–Hochberg False Discovery Rate (*q* < 0.05) was used. In order to look for specific comparisons of interest a *post-hoc* Tukey's Honest Significant Difference (HSD) test was used after the ANOVA.

#### 2.8.2 AIMS and RT-PCR data analysis

Total AIMS data at 1, 7, 14 and 21 days were analyzed using a two-way ANOVA with Šídák's multiple comparisons test. Individual AIMS components on day 22 were analyzed using Kruskal–Wallis test followed by Dunn's Multiple Comparison Test. RT-PCR data were analyzed using a one-way ANOVA with Bonferroni's Multiple Comparison Test. Significance was set at *P* < 0.05. Analysis was performed using Prism 5 (GraphPad, La Jolla, CA).

## 3 Results

### 3.1 Characterization of 6-OHDA lesion of the nigrostriatal pathway and abnormal involuntary movements following chronic treatment with LDOPA in Drd1 and Drd2 EGFP-L10a Bac mice

Successful lesion of the nigrostriatal pathway was confirmed both behaviorally and histologically. Thus, 14 days post-lesion, all animals were assessed for rotational bias by measuring spontaneous full rotations contraversive and ipsiversive to the lesioned hemisphere over a 10 min period in glass cylinders. All animals included in the study displayed a ≥90% ipsiversive rotational bias (see Supplementary Table 3). Post-mortem, lesion efficiency was assessed by calculating nigral tyrosine hydroxylase (TH) immunoreactive cell loss. All animals included in the study had a ≥90% loss of tyrosine hydroxylase immunoreactive cells in the lesioned hemisphere as compared to the intact hemisphere (see Supplementary Table 3).

A timecourse illustrating the development of AIMS over the 21 day treatment period is shown in Supplementary Figure 1A. On day 22, animals from all 4 treatment groups exhibited AIMS. However, both DRD1A and DRD2 animals treated with chronic LDOPA exhibited significantly higher levels of AIMs compared to animals in the acute treatment group who had been treated with saline for 21 days (Supplementary Figures 1B–D). No orofacial AIMS were observed therefore these data are not shown. There was no significant difference in the total of peak dose AIMs between Drd1 and Drd2 EGFP-L10a Bac mice.

### 3.2 Striatal gene expression analysis following acute or chronic treatment with LDOPA in DRD1A and DRD2 EGFP-L10a Bac mice

**Figure 1:**
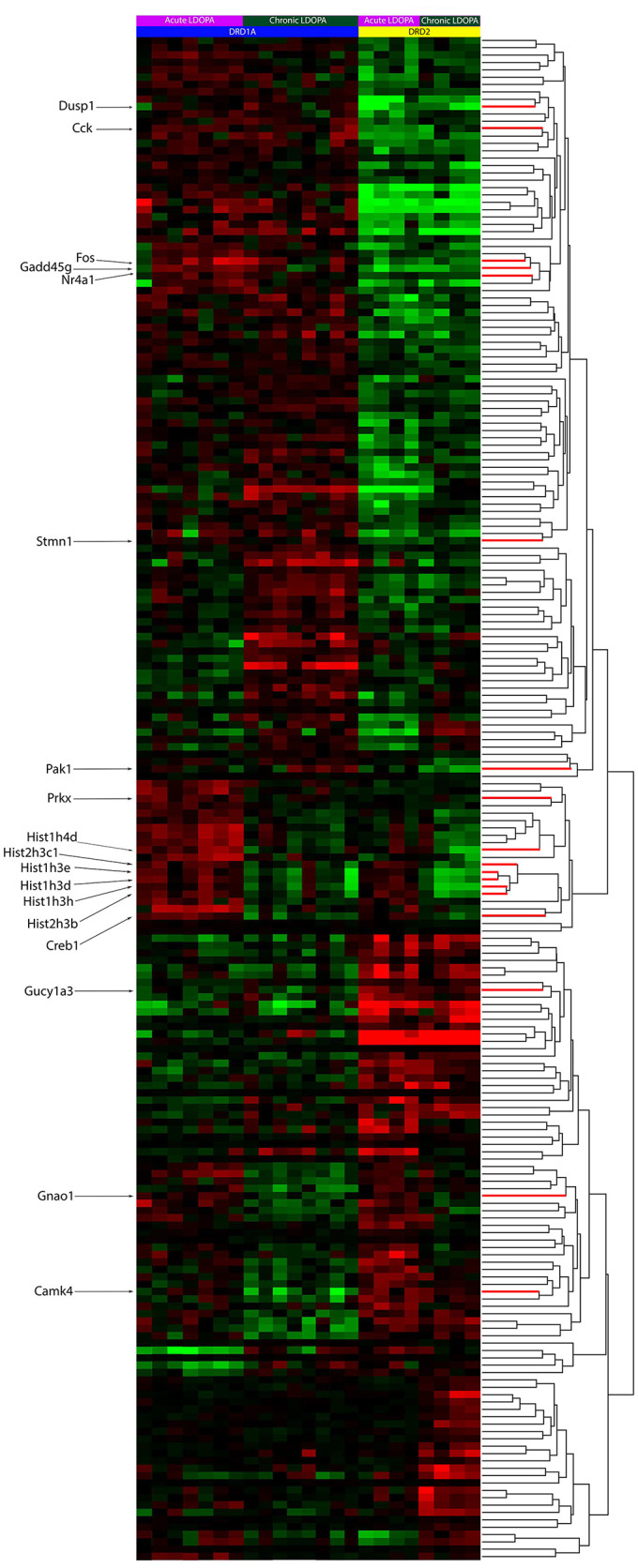
Heat-map of supervised clusters of gene expression changes in Drd1a and Drd2 EGFP-L10a expressing MSNs following acute or chronic LDOPA in a mouse model of PD. A two-way hierarchical clustering of the 207 significantly varying probes, as determined through a Benjamini–Hochberg FDR corrected ANOVA (*q* < 0.05), is presented. Color indicates direction of and magnitude of log_2_ fold-change (red, increased; green, decreased; black, no significant change). The dendrogram to the right shows the hierarchical clustering based on similarity between the expression of genes. Specific genes of interest are named on the left. Within each treatment group, animals are presented as those with the lowest AIMS score **(left)** to those with the highest AIMS score **(right)**. The number of animals per groups was as follows: Acute LDOPA Drd1a (7), acute LDOPA Drd2 (8), chronic LDOPA Drd1a (4), chronic LDOPA Drd2 (4). The number of sample replicates was 1.

To find the genes that statistically varied with the greatest confidence between sample groups, a one-way ANOVA with a Benjamini-Hochberg False Discovery Rate (*q* < 0.05) was used. In total, 207 probes representing 195 unique genes or transcripts were found with this test (Supplementary Table 4). The results are clustered in Figure 1 for visualization purposes. Importantly, among the genes found, we were able to replicate known changes in the expression of *PDYN, HTR2A*, and *CREB1* following chronic LDOPA (Supplementary Table 4), validating the use of our experimental paradigm to identify differences in the molecular responses of the direct and indirect pathway to chronic LDOPA.

**Figure 2:**
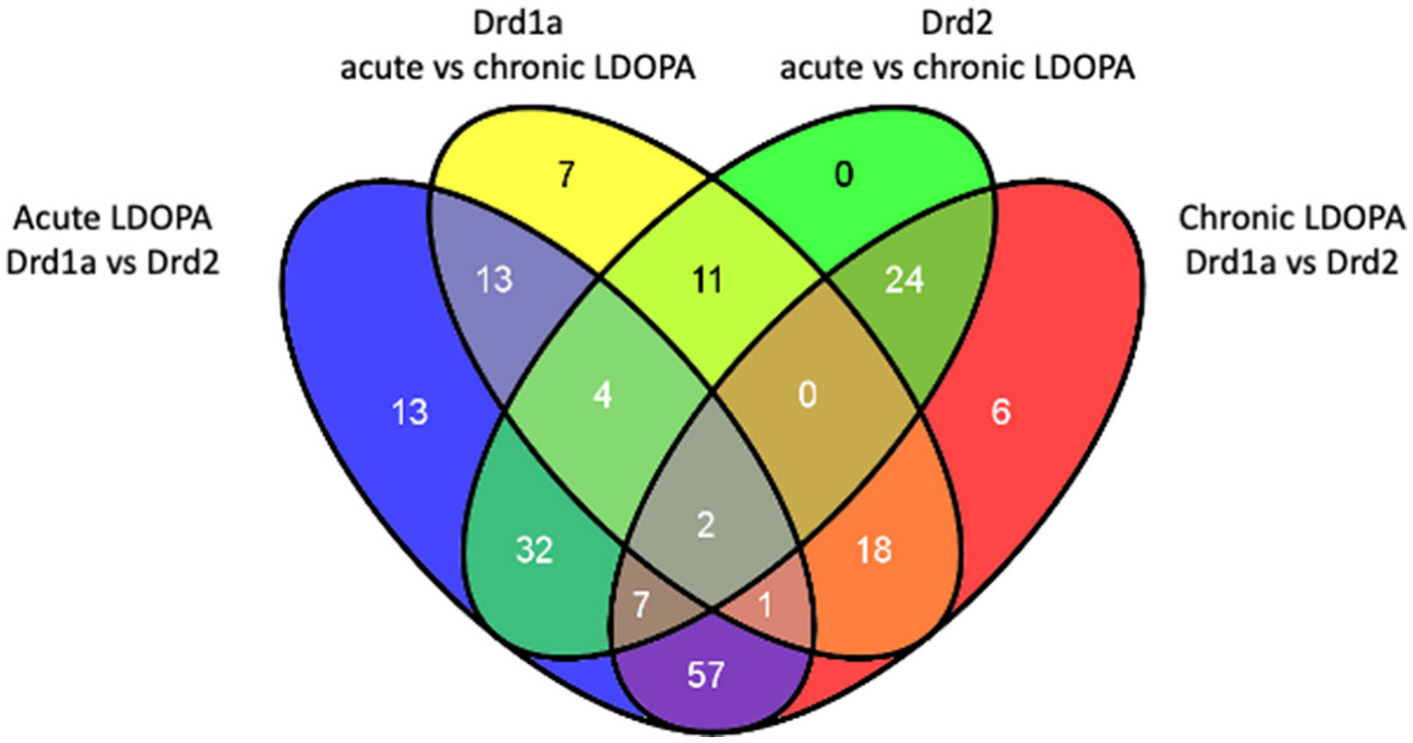
Overlap of differentially expressed genes in Drd1a and Drd2 EGFP-L10a expressing MSNs following acute and chronic LDOPA in a mouse model of PD. Venn diagram showing the total number of differentially expressed genes and their overlap between the four experimental groups for statistically significant changes [Benjamini–Hochberg FDR corrected ANOVA (*q* < 0.05)]. Acute LDOPA Drd1a vs. Drd2: Genes altered between Drd1a and Drd2 MSNs in animals treated with acute LDOPA. Drd1a acute vs. chronic LDOPA: Genes altered in Drd1a MSNs following acute vs. chronic LDOPA treatment. Drd2 acute vs. chronic LDOPA: Genes altered in Drd2 MSNs following acute vs. chronic LDOPA treatment. Chronic LDOPA Drd1a vs. Drd2: Genes altered between Drd1a and Drd2 MSNs after chronic LDOPA treatment.

A *post-hoc* Tukey's HSD test was applied to the 207 probes of interest to perform select pairwise comparisons. Probes altered between DRD1A and DRD2 MSNs in animals treated with acute LDOPA are found in Supplementary Table 5. Probes altered in DRD1A MSNs following acute vs. chronic LDOPA treatment are found in Supplementary Table 6. Probes altered in DRD2 MSNs following acute vs. chronic LDOPA treatment are found in Supplementary Table 7. Probes altered between DRD1A and DRD2 MSNs after chronic LDOPA treatment are found in Supplementary Table 8. Venn analysis was used to generate lists of gene expression changes both common to and exclusive to Drd1 and DRD2 EGFP-L10a expressing MSNs following acute and chronic LDOPA treatment (Figure 2). The largest dissimilarity was between the DRD1A and DRD2 EGFP-L10a expressing MSNs, followed by animals treated with acute vs. chronic LDOPA. Thus, 129 genes were differentially expressed when comparing DRD1A and DRD2 EGFP-L10a Bac mice treated with acute LDOPA, 67 of which remained differentially expressed between DRD1A and DRD2 EGFP-L10a Bac mice treated with chronic LDOPA (Figure 2). In contrast, in DRD1A EGFP-L10a Bac mice 56 genes were differentially expressed when comparing animals treated with acute vs. chronic LDOPA, and in DRD2 EGFP-L10a Bac mice 80 genes were differentially expressed when comparing animals treated with acute vs. chronic LDOPA (Figure 2). Finally, 115 genes were differentially expressed when comparing DRD1A and DRD2 EGFP-L10a Bac mice treated with chronic LDOPA, 48 of which were not differentially expressed when comparing DRD1A and DRD2 EGFP-L10a Bac mice treated with acute LDOPA (Figure 2).

### 3.3 Pathways altered in DRD1A and DRD2 EGFP-L10a expressing MSNs following acute and chronic treatment with LDOPA

The 195 unique genes that were differentially expressed among the four experimental groups were subjected to Enrichr analysis, which revealed several pathways differentially altered in DRD1A and DRD2 EGFP-L10a expressing MSNs following acute and chronic LDOPA as well as some common to both pathways (Table 1).

**Table 1:** KEGG defined pathways and their associated deregulated genes in Drd1a and Drd2 expressing EGFP-L10a MSNs following acute or chronic LDOPA in a mouse model of PD.

**KEGG term**	***P*-value**	***Z*-score**	**Combined score**	**Genes**
**Acute LDOPA Drd1a vs. Drd2**				
HSA04010_MAPK_SIGNALING_PATHWAY	0.0001	−2.16	21.42	PRKX; GADD45G; DUSP1; DUSP6; STMN1; NR4A1; FOS; RASGRP4; RASGRP2
HSA04742_TASTE_TRANSDUCTION	0.0066	−1.96	9.85	PRKX; GNG13; PDE1A
HSA04740_OLFACTORY_TRANSDUCTION	0.0217	−1.70	6.53	PRKX; GNAL
HSA04540_GAP_JUNCTION	0.0322	−1.80	6.17	PRKX; DRD2; GUCY1A3
**Drd1a acute vs. chronic LDOPA**				
HSA04916_MELANOGENESIS	0.0002	−1.97	17.11	PRKX; DVL3; GNAO1; CREB1
HSA04020_CALCIUM_SIGNALING_PATHWAY	0.0012	−1.91	12.77	PRKX; CAMK4; GNAL; HTR2A
HSA04740_OLFACTORY_TRANSDUCTION	0.0039	−1.70	9.46	PRKX; GNAL
HSA04720_LONG_TERM_POTENTIATION	0.0171	−1.70	6.90	PRKX; CAMK4
HSA04540_GAP_JUNCTION	0.0326	−1.74	5.95	PRKX; HTR2A
**Drd2 acute vs. chronic LDOPA**				
HSA04510_FOCAL_ADHESION	0.0082	−2.17	10.43	FARP2; MYL9; ITGA9; PAK1
HSA05120_EPITHELIAL_CELL_SIGNALING_IN_HELICOBACTER_PYLORI_INFECTION	0.0325	−1.79	6.13	PAK1; ATP6V0A2
HSA04730_LONG_TERM_DEPRESSION	0.0396	−1.81	5.85	GUCY1A3; GNAO1
**Chronic LDOPA Drd1a vs. Drd2**				
HSA04010_MAPK_SIGNALING_PATHWAY	0.0004	−2.16	16.97	GADD45G; DUSP1; STMN1; NR4A1; FOS; PAK1; RASGRP2
HSA04540_GAP_JUNCTION	0.0174	−1.91	7.75	DRD2; GUCY1A3; HTR2A
HSA00190_OXIDATIVE_PHOSPHORYLATION	0.0343	−1.74	5.87	COX5A; NDUFA3; ATP6V0A2

Enrichr (http://amp.pharm.mssm.edu/Enrichr/) was applied to identify significant KEGG defined pathways associated with the sets of differentially expressed genes in Drd1a and Drd2 expressing MSNs following acute or chronic LDOPA.

The most significantly implicated pathway was the mitogen-activated protein kinase (MAPK) cascade (KEGG # HSA04010). Specifically, the significant MAPK pathway-associated genes *NR4A1, GADD45G, STMN1, FOS*, and *DUSP1*, were upregulated in the DRD1A expressing MSNs compared to DRD2 EGFP-L10a expressing MSNs in animals receiving both acute LDOPA (Enrichr *P*-value 0.0001) and chronic LDOPA (Enrichr *P* value 0.0004). Likewise, the Gap junction pathway (KEGG # HSA04540) was also found to be associated when comparing DRD1A and DRD2 EGFP-L10a expressing MSNs following both acute and chronic LDOPA, with the former being driven by changes in the expression of *PRKX*, DRD2 and *GUCY1A3* (Enrichr *P* value 0.0332) and the latter by DRD2, *GUCY1A3* and *HTR2A* (Enrichr *P* value 0.0174; Table 1).

DRD1A EGFP-L10a expressing MSNs had an overrepresentation of the long-term potentiation pathway (LTP) (KEGG # HSA04720; Enrichr *P* value 0.0171) after chronic treatment with LDOPA, whereas as DRD2 EGFP-L10a expressing MSNs had significant involvement of long-term depression (LTD) (KEGG # HSA04730; Enrichr *P* value 0.0396) (Table 1). The LTP cascade was mainly driven by down-regulation of *PRKX* (KEGG #5613) and *CAMK4* (KEGG #814) in DRD1A EGFP-L10a expressing MSNs, with LTD in DRD2 EGFP-L10a expressing MSNs driven by upregulation of *GUCY1A3* (KEGG #2982) and downregulation of *GNAO1* (KEGG #2775).

### 3.4 Validation of findings

**Figure 3:**
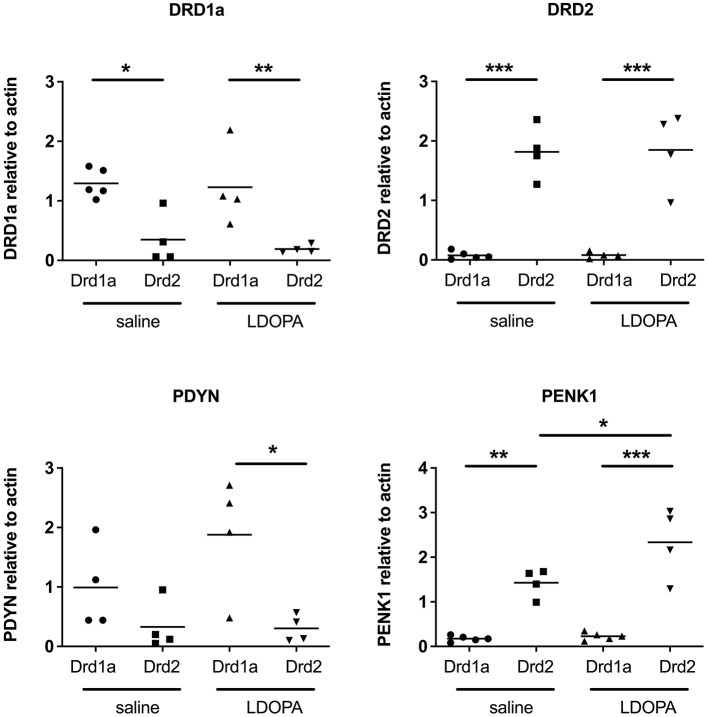
Q-RTPCR validation of known gene expression patterns in Drd1a and Drd2 EGFP-L10a expressing MSNs following acute or chronic treatment with LDOPA in a mouse model of PD. Individual values for each gene of interest are presented for each animal relative to actin. *P < 0.05, **P < 0.01, ***P < 0.001, One way ANOVA with Bonferroni's Multiple Comparison Test.

To validate our findings, qRT-PCR was performed on eight selected genes. First, the use of TRAP to successfully differentiate the MSNs of the direct and indirect pathways was confirmed by the selective enrichment of DRD1A and prodynorphin (*PDYN*) in RNA extracted from DRD1A EGFP-L10a Bac mice and of DRD2 and preproenkephalin 1 (*PENK1*) in RNA extracted from DRD2 EGFP-L10a Bac mice (Figure 3).

**Figure 4:**
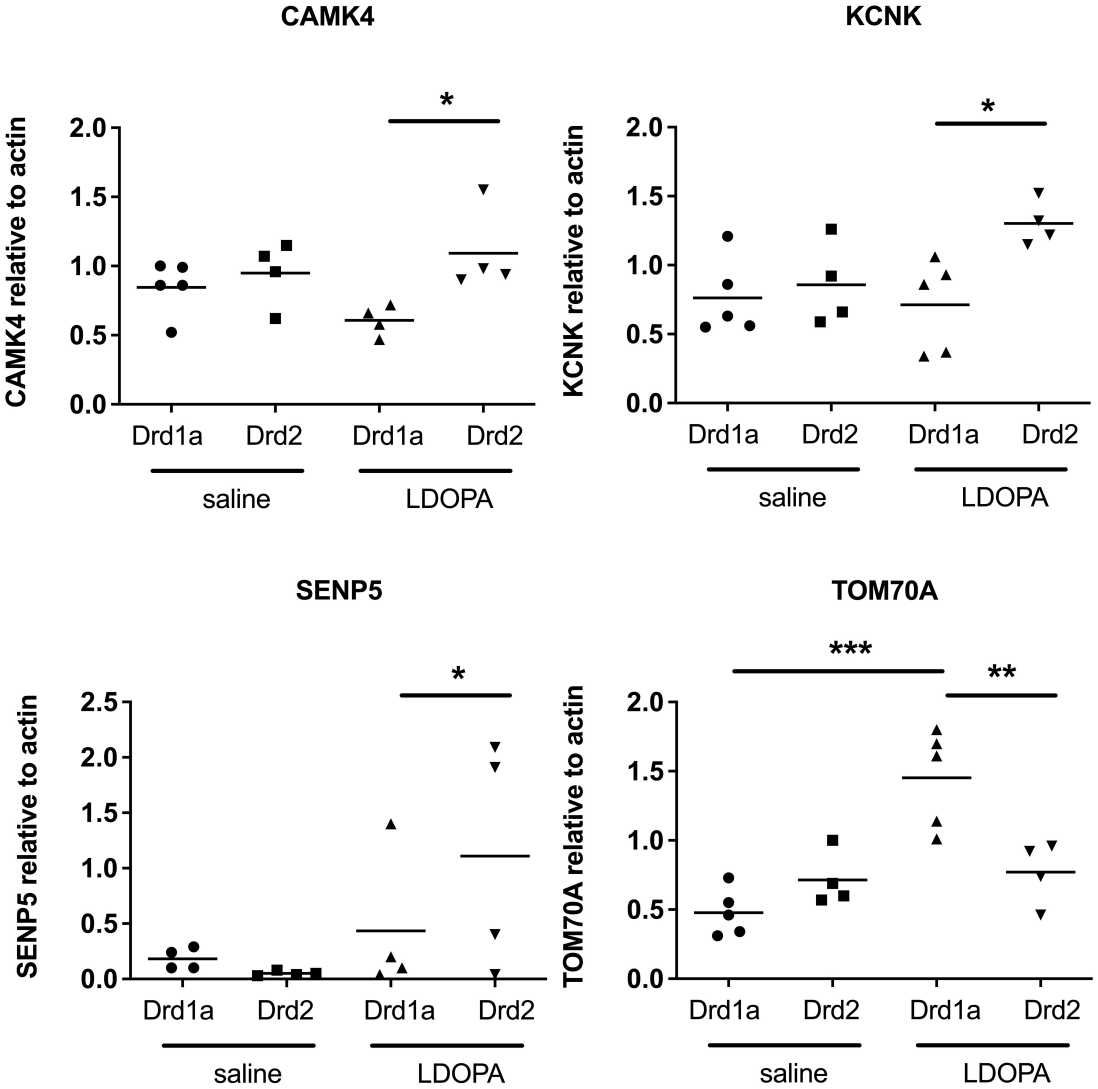
Q-RTPCR validation of gene expression patterns identified using microarray in Drd1a and Drd2 EGFP-L10a expressing MSNs following acute or chronic treatment with LDOPA in a mouse model of PD. Individual values for each gene of interest are presented for each animal relative to actin. *P < 0.05, **P < 0.01, ***P < 0.001, One way ANOVA with Bonferroni's Multiple Comparison Test.

Second, we chose four genes identified in the microarray as being differentially expressed across our four experimental groups to validate using qRT-PCR. Results of the qRT-PCR confirmed the microarray data. Thus, transcripts for *CAMK4, KCNK* and *SENP5* were all significantly upregulated and *TOM70A* was significantly downregulated in DRD2 EGFP-L10a expressing MSNs compared to DRD1A EGFP-L10a expressing MSNs following chronic treatment with LDOPA (Figure 4).

## 4 Discussion

Here, we describe translational gene expression changes in a mouse model of LID, employing a powerful methodology that allows for the selective study of DRD1A expressing striatonigral and DRD2 expressing striatopallidal projection MSNs. We have identified 195 unique genes altered in these two distinct neuronal subpopulations, implicated in the pathogenesis of LID, the majority of which are novel. Furthermore, pathway/GO analysis has revealed several differentially altered molecular pathways in which the gene expression changes were organized.

### 4.1 Mitogen-activated protein kinase pathway and LID

The mitogen-activated protein kinase (MAPK) cascade, was the most significantly implicated GO pathway differentiating the DRD1A expressing (direct pathway) and DRD2 expressing (indirect pathway) MSNs, following both acute and chronic LDOPA treatment. The specific MAPK pathway related genes in our study included *DUSP1, STMN1, GADD45G, FOS* and *NR4A1*, all of which were upregulated in the MSNs of the direct pathway compared to the indirect pathway. This finding confirms previous observations for MAPK cascade activation in striatonigral DRD1A expressing MSNs, where an increase in *ERK* and *MSK1* phosphorylation was observed in dyskinetic mice in response to chronic LDOPA treatment (Santini et al., 2009) as well as a second study which, using the MAPK pathway gene *FOS*, combined optogenetics with the FosTRAP method and found that primarily direct pathway MSNs are activated during LID (Girasole et al., 2018).

DUSP1 is a phosphatase that dephosphorylates MAPK and suppresses activation of MAPK by oncogenic *RAS* (Wancket et al., 2012). A previous study found that *DUSP1* and *DUSP6* transcripts are upregulated in hemiparkinsonian mice subjected to LDOPA treatment (Pérez-Sen et al., 2019). Our data extend this finding and suggest this upregulation is specific to MSNs of the direct pathway. *STMN1* is a downstream effector of MAPK signaling via a miRNA-regulated mechanism and a potential oncogene in melanoma (Feng et al., 2017; Chen et al., 2013). *GADD45G* mediates activation of p38/JNK via MTK1/MEKK4 kinase (Balliet et al., 2003). *FOS* is one of the transcription factors regulated by MAPK signaling (Yokoyama et al., 2013). *NR4A1* encodes an intracellular transcription factor (Maxwell and Muscat, 2006). The involvement of these four genes in PD or LID has not been previously reported, although a previous study reported that FOSB and NURR1 (encoded by *NR4A2*) protein are elevated in dyskinetic rats (Steece-Collier et al., 2020). Elevated levels of FOSB have also been reported in dyskinetic non-human primates (Beck et al., 2019).

Notably, we observed that chronic treatment with LDOPA had opposing effects on the expression of PAK1, a known activator of the MAPK pathway and a breast cancer oncogene (Shrestha et al., 2012). Thus, *PAK1* gene expression was downregulated in the indirect pathway and upregulated in the direct pathway after chronic LDOPA treatment. As both the direct and indirect pathways play an important role in LID, this bidirectional alteration in gene expression make PAK1 particularly interesting. In addition to effects on MAPK, PAK1 is also a regulator of LTP via modulation of the actin cytoskeleton, with actin being heavily involved in receptor trafficking (Asrar et al., 2009). Altered subcellular trafficking of glutamatergic NMDA receptors has been implicated in LID, secondary to changes in the NR2A/NR2B subunit composition due to decreased NR2B levels resulting from deficient anchoring in the post-synaptic density (Fiorentini et al., 2006; Cattabeni et al., 1999). Given our observations future experiments might investigate the potential role of PAK1 in aberrant receptor trafficking in LID, with a view to potential therapeutic interventions.

### 4.2 Cyclic AMP response-binding protein and LID

In our study, chronic LDOPA caused a downregulation of CREB1 RNA in both the direct and indirect pathways. Our observed implication of CREB1 in LID is consistent with another study using a transcriptomic approach, that reported that CREB1 was among the top upstream regulatory transcription factors implicated in rats with LID (Dyavar et al., 2020). In further support of a role for CREB1 in LID, Riluzole, known to reduce the activity of CREB1, has been shown to weaken LID in rats (Pagliaroli et al., 2019). It is widely understood that ERK and MAPK signaling regulate neuronal CREB transcription through phosphorylation (Koga et al., 2019) and previous studies have found that increased CREB phosphorylation is associated with LID as it is a downstream target of MSK1 (itself a target of ERK) (Azkona et al., 2014; Reyskens and Arthur, 2016). Administration of mGlu5R antagonists have been associated with reduced striatal levels of phosphorylated ERK and MSK1 as well as weakened dyskinesia in experimental PD (Spigolon and Fisone, 2018; Sebastianutto et al., 2020; Rylander et al., 2009). Our findings provide further support for the role of CREB1 in both the direct and indirect pathways in LID. However, as CREB1 plays a key role in regulating the balance between excitatory and inhibitory signaling in the brain (Chowdhury et al., 2023), it is certainly possible that the observed alterations in CREB1 in the present study are a homeostatic adaptation to limit excessive signaling within striatal MSNs. Given this possibility, clearly further studies are certainly required to investigate the potential of manipulating this pathway as a possible treatment for LID.

### 4.3 Histone cluster proteins and LID

Another target of ERK/MSK1 signaling is histone H3 and an increase in its phosphorylation has been reported in LID (Ciccarelli and Giustetto, 2014). In support of this, we found several histone cluster proteins (HIST1H3D, HIST1H4D, HIST1H3E, HIST1H3H, HIST2H3C1, and HIST2H3B) to be downregulated in both the direct and indirect pathways following chronic treatment with LDOPA. Indeed, previous studies have found that long lasting cellular adaptations in animal models of LID include histone modifications through lysine acetylation, lysine and arginine methylation, serine and threonine phosphorylation, lysine ubiquitination and sumoylation, and the activation of histone kinases, histone acetyltransferases, histone deacetylases, and histone methyltransferases (reviewed in Cenci and Konradi, 2010). Such alterations affect the histone-DNA interaction as well as the ability of transcription factors to bind. We are not aware of any studies targeting modifications to histone cluster proteins as a potential therapeutic intervention in LID thus this would make a novel future direction.

### 4.4 LTP, LTD, and LID

Another major finding of our study was the involvement of calcium signaling with overlapping involvement of LTP in the direct pathway, and LTD in the indirect pathway in LID. Maladaptive plasticity in the striatal MSNs of the direct and indirect pathways in PD and LID has been previously described. Thus, in animal models of PD, corticostriatal LTP is lost but can be restored by treatment with LDOPA (Picconi et al., 2012; Calabresi et al., 2007; Costa et al., 2012). Interestingly, following induction of LTP, low-frequency stimulation can reverse the effect (depotentiation) but this ability is selectively lost in animals with LID (Picconi et al., 2012). These observations have led to the suggestion that striatal MSNs are able to manifest homeostatic adaptations in the number of excitatory corticostriatal synapses and intrinsic excitability in response to perturbations in dopamine signaling (Fieblinger et al., 2014).

In the present study, the significant implication of calcium signaling and LTP cascades in the direct pathway were mainly driven by down-regulation of PRKX and CAMK4 in chronic LDOPA treated animals compared to acute LDOPA treated animals. Both PRKX and CAMK4 are involved in LTP (Anderson and Kane, 1998), with calcium-dependent nuclear signaling via CAMK4 and CREB being involved in LTP-associated NMDA receptor post synaptic density-95 blockade (Bell et al., 2013). While CAMK2 has been extensively implicated in NMDAR-dependent LTP, CAMK4 is less well studied in this context (Lisman et al., 2012). CAMK4 has been shown to regulate Ca-dependent transcription via phosphorylation of various transcription factors including CREB (Enslen et al., 1995). PRKX is a cAMP-dependent protein kinase similar to PKA, a downstream target of DRD1A receptor activation which is a requirement for LTP (Li, 2011). It remains to be seen what role CAMK4 and PRKX might play in LTP in the context of LID and whether they are involved in the reported loss of depotentiation. In the indirect pathway, an upregulation of GUCY1A3 and downregulation of GNAO1 underscored the involvement of LTD. GUCY1A3 encodes the alpha-3 subunit of soluble guanylate cyclase (sGC) an enzyme that is crucial for the production of cyclic guanosine monophosphate (cGMP) in response to binding of nitric oxide (NO) reviewed in De Pauw et al. (2016). LID is associated with a loss of corticostriatal LTD (De Pauw et al., 2016) and NO has been demonstrated to mediate a cGMP-dependent LTD in striatal MSNs reviewed in Zhai et al. (2019). Thus, although the precise mechanism by which GUCY1A3 may influence LID remains to be resolved, it appears likely that GUCY1A3 may mediate these effects by modulating the production of cGMP in NO-dependent LTD.

Interestingly, we also found that CCK (cholecystokinin) was upregulated in the direct vs. indirect pathway in animals treated with both acute LDOPA or chronic LDOPA. Activation of the central CCK receptor (CCK-B) has been shown to augment LTP in guinea pig hippocampus (Yasui and Kawasaki, 1995). Furthermore, the CCK analog CCK-8S has been shown to inhibit LID in parkinsonian squirrel monkeys (Boyce et al., 1990). While the striatum has been shown to have high concentrations of CCK and abundantly expresses CCK receptors, the role of CCK in dopaminergic regulation of LTP and its relationship with the mechanism by which it may inhibit LID has not been studied and thus represents a new avenue of research (Okonkwo and Adeyinka, 2019).

### 4.5 Comparison to previous studies in DRD1A and DRD2 EGFP-L10a expressing BacTRAP animals

In 2014, Heiman et al. performed a study in DRD1A and DRD2 EGFP-L10a expressing BacTRAP animals, exploring alterations in gene expression resulting from dopamine denervation and in response to treatment with two different doses of LDOPA for 9 days (Heiman et al., 2014a). This study noted only a small number of gene expression changes in the indirect pathway in response to LDOPA, whereas the direct pathway exhibited profound alterations in CREB, AP-1 and ERK-mediated signaling, with many of the associated genes correlating with the dose of LDOPA. Although differences in the experimental paradigms employed in the present study and the work of Heiman et al., preclude the direct comparison of the datasets, importantly, there is some overlap in the pathways identified, notably alterations in MAPK signaling in the direct pathway. Our study is distinct from the Heiman et al., study in two critical ways. First, Heiman et al., focussed on genes implicated with dopamine depletion and the *severity* of LID and investigated the effects of chronic treatment with high dose LDOPA vs. low dose LDOPA, whereas our study examined the effects of *chronic* LDOPA treatment vs. *acute* LDOPA (single dose). The rationale for our treatment regimen was to identify changes in gene expression resulting from repeated vs. initial exposure to LDOPA as these genes might best reflect pathological processes implicated in the development of LID in response to repeated exposure. Second, we examined tissues collected 40 min after the last dose of LDOPA, whereas Heiman et al., examined tissues 3 h and 20 min after the last LDOPA dose. Although we have not performed a pharmacokinetic analysis in the present study, the use of LDOPA in 6-OHDA lesioned mice is extensively described in the literature and it is well established that LDOPA reaches peak plasma concentrations 30–60 min post administration (Lundblad et al., 2004; Cenci and Crossman, 2018; Putterman et al., 2007; Eriksson et al., 1984; Winkler et al., 2002). After this point, LDOPA declines in its bioavailability and returns to control values ~120 min post administration (Kääriäinen et al., 2012; Peng et al., 2019; Spencer and Wooten, 1984). Thus, in contrast to Heiman et al., our study is designed to capture gene expression concurrent with the expression of LID. Accordingly, there are meaningful differences between the two studies that have a clear impact on the hypothesis being tested and are reflected in the different genes and pathways identified in the two pieces of work.

### 4.6 Potential caveats

The present work needs to be considered in light of some caveats. First, striatal DRD2 expression is not entirely exclusive to GABAergic MSNs. Indeed, a comprehensive analysis of EGFP expression in DRD1a and DRD2-BacTRAP mice demonstrated that while in DRD1A-BacTRAP mice striatal EGFP expression was restricted to striatonigral MSNs of the direct pathway, DRD2-BacTRAP mice showed expression in striatopallidal neurons of the indirect pathway as well as in cholinergic interneurons (Valjent et al., 2009). Therefore, it is feasible that the observed gene expression changes in the striatum of DRD2-BacTRAP animals reflect some degree of alterations in cholinergic interneurons. As cholinergic interneurons have themselves been implicated in the pathophysiology of LID, this is worthy of consideration. With respect to the pathways implicated in the present study, dopaminergic control of corticostriatal LTD in MSNs has been shown to be influenced by cholinergic interneurons (Wang et al., 2006). Thus, our observation of the involvement of LTD in the indirect pathway may reflect a contribution from cholinergic interneurons. The relative contribution of transcripts isolated from cholinergic interneurons in our study is unknown, however it should be noted that GABAergic MSNs comprise 95% of striatal cells, with cholinergic interneurons making up the remaining 5% of cells (Kawaguchi, 1997; Tepper and Bolam, 2004). Second, our data are restricted to those that manifest in alteration of RNA expression levels and therefore do not detect LID-related processes that involve enzymatic reactions (e.g. phosphorylation) or protein-protein interactions. For example, mTOR signaling has been shown to be a downstream target in the MAPK pathway associated with LID, however as this observation is associated with signaling events, not a change in expression *per se*, this was not detected in our study (Zhu et al., 2020; Jin et al., 2010). Second, a recent study reported that RNAseq increased the sensitivity of the TRAP approach by ~10-fold compared to the microarray approach employed here, suggesting that had TRAP-RNAseq been used additional genes of interest may have been found (Montalban et al., 2022). Indeed, the TRAP-RNAseq model has led to the discovery of previously unknown distinct MSN subpopulations involved in locomotor control (Fieblinger, 2021). Third, while our study focused on changes in gene expression in the striatum, other studies have demonstrated that chronic LDOPA can induce changes in gene expression in other areas of the brain such as the frontal cortex, as well as in cell types other than MSNs, including immune and endothelial cells (Radlicka et al., 2021). Lastly, our study did not consider changes in DNA methylation which have been implicated in the development and maintenance of LID (Figge et al., 2016).

## 5 Conclusion

**Figure 5:**
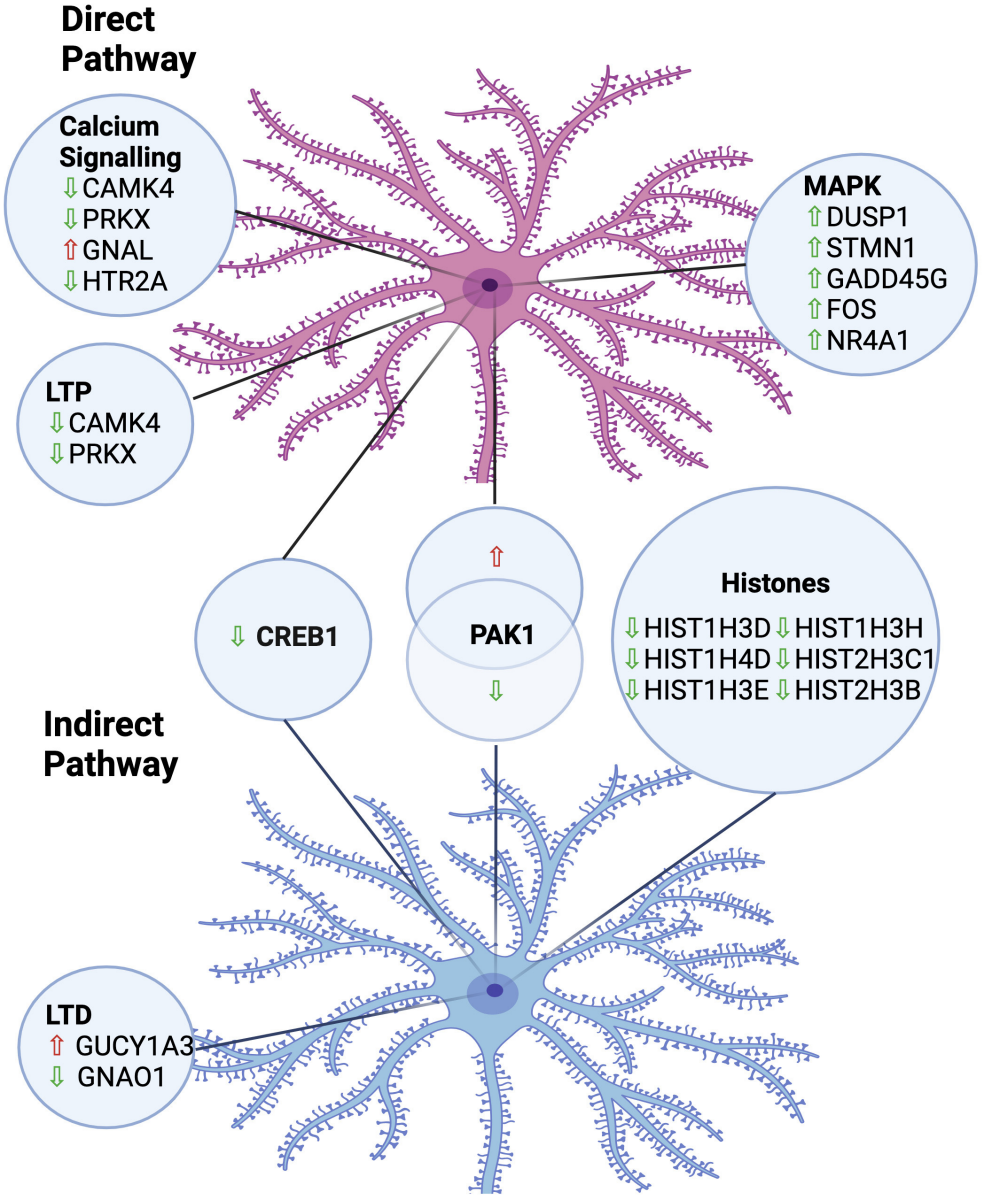
Summary of implicated pathways and associated gene expression changes in MSNs of the direct and indirect pathways in an animal model of LDOPA-induced dyskinesia. LTP, Long-Term Potentiation; LTD, Long-Term Depression; Upward arrow, Upregulated; Downward arrow, Downregulated. Created in https://BioRender.com.

In summary, we have employed a powerful experimental paradigm, combining BacTRAP (to selectively isolate RNA from either DRD1A expressing striatonigral or DRD2 expressing striatopallidal MSNs), with a widely used rodent model of LID, to reveal changes in translational gene expression following repeated LDOPA treatment, summarised in Figure 5. Our findings have revealed several novel translational changes implicated in LID. Our data highlight the involvement of several genes in the MAPK pathway, calcium signaling and LTP/LTD in maladaptive responses to chronic treatment with LDOPA. These findings have implicated the potential of targeting these genes and pathways as novel therapeutic interventions to prevent the development of LID, a critical unmet need in the treatment of PD.

## Data Availability

The original contributions presented in the study are publicly available. This data can be found here: https://www.ncbi.nlm.nih.gov/geo/query/acc.cgi?acc=GSE291024.

## Supplementary material

Supplementary Figure 1 Abnormal involuntary movements (AIMs) in 6-OHDA lesioned Drd1a and Drd2 EGFP-L10a expressing mice following acute or chronic treatment with LDOPA. **(A)** Total AIMS observed over a period of 120 min 1, 7, 14 and 21 days post treatment with vehicle or LDOPA methyl ester/benserazide (6/15 mg/kg). ^*^*P* < 0.05, ^***^*P* < 0.001 *cf* day 1, Two way ANOVA with Šídák's multiple comparisons test. **(B–D)** On day 22, AIMs were assessed in all animals for a period of 1 min, 40 min post treatment with LDOPA methyl ester/benserazide (6/15 mg/kg). **(B)** Limb AIMS, **(C)** Axial AIMS, **(D)** Total AIMS. ^*^*P* < 0.05, ^***^*P* < 0.001 Kruskal–Wallis test followed by Dunn's Multiple Comparison Test.
