# Supplementary figures and images for: Translational profiling reveals novel gene expression changes in the direct and indirect pathways in a mouse model of levodopa induced dyskinesia
Source: Front Cell Neurosci. 2025 Mar 12;18:1477511. doi: 10.3389/fncel.2024.1477511 (PMC11936753; doi:10.3389/fncel.2024.1477511)

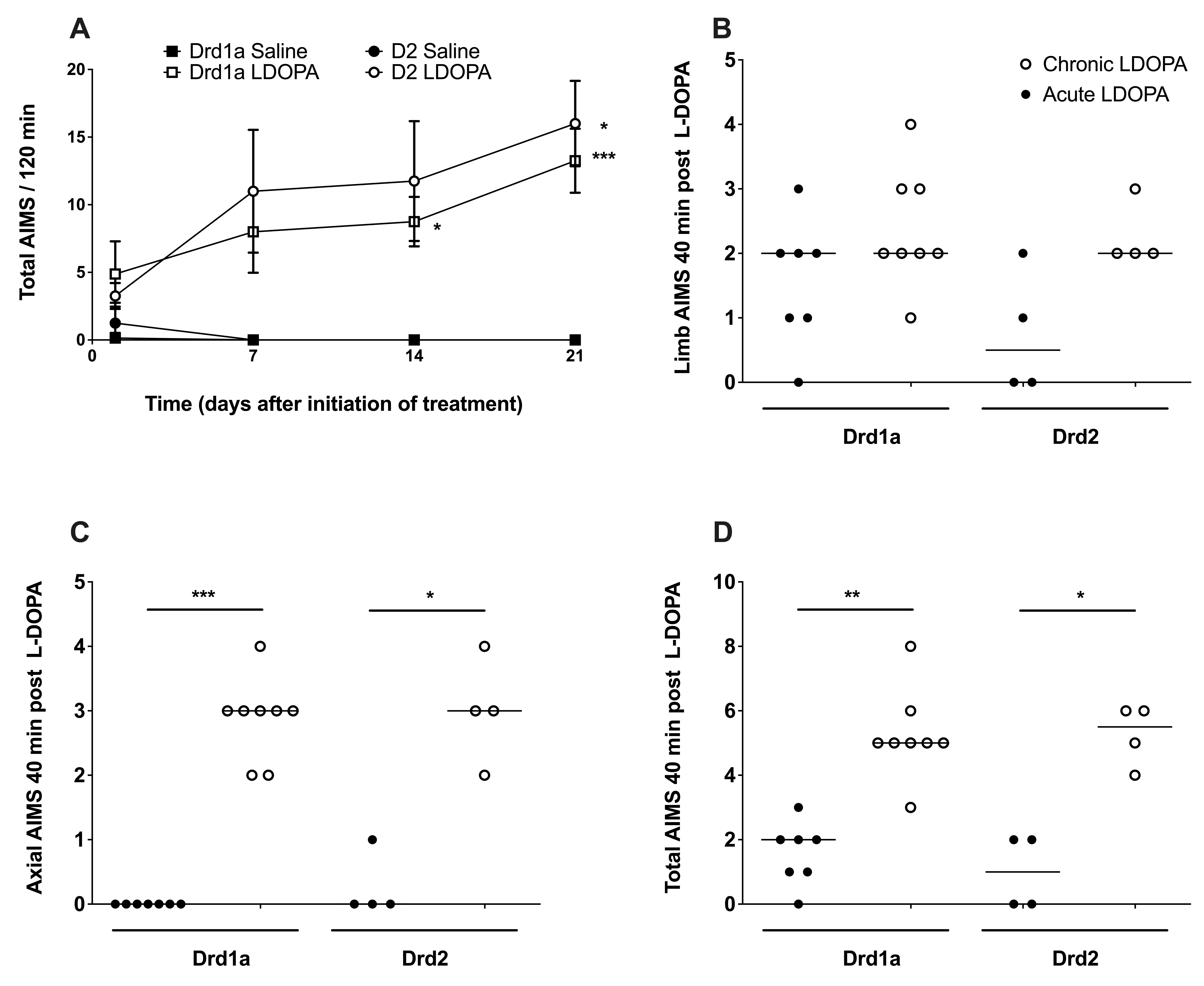

Supplement: Supplementary Figure 1 — Abnormal involuntary movements (AIMs) in 6-OHDA lesioned Drd1a and Drd2 EGFP-L10a expressing mice following acute or chronic treatment with LDOPA. (A) Total AIMS observed over a period of 120 min 1, 7, 14 and 21 days post treatment with vehicle or LDOPA methyl ester/benserazide (6/15 mg/kg). *P < 0.05, ***P < 0.001 cf day 1, Two way ANOVA with Šídák's multiple comparisons test. (B–D) On day 22, AIMs were assessed in all animals for a period of 1 min, 40 min post treatment with LDOPA methyl ester/benserazide (6/15 mg/kg). (B) Limb AIMS, (C) Axial AIMS, (D) Total AIMS. *P < 0.05, ***P < 0.001 Kruskal–Wallis test followed by Dunn's Multiple Comparison Test. [file Image_1.JPEG]
